# Sensory neuropathy and metabolic risk factors in human immune deficiency virus infected South Africans receiving protease inhibitors

**DOI:** 10.1186/s12981-015-0073-8

**Published:** 2015-09-23

**Authors:** John-Randel Vermaak, Joel A. Dave, Naomi Levitt, Jeannine M. Heckmann

**Affiliations:** Neurology Research Group, Division of Neurology, Department of Medicine, University of Cape Town, Cape Town, South Africa; Division of Endocrinology and Diabetic Medicine, Department of Medicine, University of Cape Town, Cape Town, South Africa

**Keywords:** Lopinavir, Ritonavir, Distal sensory polyneuropathy, Metabolic, Triglycerides, Dysglycemia, Protease inhibitors, African, Symptomatic neuropathy, Anti-retroviral therapy

## Abstract

**Background:**

Protease inhibitors (PI)s have been associated with distal sensory polyneuropathy (DSP) and metabolic complications in high-income countries. No data exist in Africans where second-line antiretroviral therapy (ART) often include PIs.

**Method:**

We performed a cross-sectional study to assess the DSP frequency and metabolic risk factors in community-based South Africans taking ritonavir-boosted lopinavir as PI. Examination findings categorized subjects as having DSP (≥1 neuropathic sign) or symptomatic DSP [DSP with symptom(s)]. Fasting-state glucose and lipid profiles were assessed. We compared the ritonavir/lopinavir-group to a nested group on first-line ART [dideoxy-nucleoside reverse transcriptase inhibitors (d-drugs)] selected from a dataset collected at the same time and matched for d-drug exposure.

**Results:**

The ritonavir/lopinavir-group (n = 86) consisted predominantly of women (84 %) with a median age of 36 years (IQR 32–41). The median current CD4+ count was 489 cells/μL (IQR 291–665). The median exposure time to ritonavir/lopinavir was 18 months (IQR 10–26) and to d-drugs, 24 months (IQR 16–38). DSP was present in 78 % and symptomatic DSP in 48 %; symptoms were most frequently of moderate intensity. Only age independently associated with DSP and symptomatic DSP (p = 0.08 and p = 0.04, respectively). None of the metabolic syndrome components showed associations with DSP or symptomatic DSP despite a trend towards hypertriglyceridemia overall. The ritonavir/lopinavir-group had less DSP compared to the d-drug only group (p = 0.002) but the frequency of symptomatic DSP was similar (p = 0.49).

**Conclusion:**

Ritonavir-boosted lopinavir did not add additional risk to developing DSP in this community-based African cohort after a median of 18 months on second-line ART.

**Electronic supplementary material:**

The online version of this article (doi:10.1186/s12981-015-0073-8) contains supplementary material, which is available to authorized users.

## Background

Distal sensory polyneuropathy (DSP) is a common neurological complication amongst people living with HIV. Reported frequencies of DSP range between 4 and 83 % depending on the population studied and the DSP definition used [1]. The clinical relevance of DSP is that a proportion of patients frequently complain of neuropathic symptoms such as pain, paresthesia or numbness in the feet which can affect their quality of life, work ability and adherence to therapies [1, 2]. Effective treatment is limited. Antiretroviral agents from the dideoxy-nucleoside reverse transcriptase inhibitor class (d-drugs) which includes stavudine and didanosine, are known to associate with the development of symptomatic DSP [1, 2].

Previously, in a cross-sectional community-based sample, we reported a high frequency of DSP among South Africans receiving first-line anti-retroviral therapy (ART), which at that time, contained stavudine. In alignment with the World Health Organization recommendations of the time [3], the South African government second-line ART regimen included ritonavir-boosted lopinavir (ritonavir/lopinavir) in combination with two nucleoside reverse transcriptase inhibitors (NRTI), stavudine or didanosine and lamivudine. Reports from North America noted associations between protease inhibitor (PI) exposure (indinavir, saquinavir, ritonavir, amprenavir, lopinavir) and DSP [4–7]. In addition, PI use among individuals from developed countries, usually older males, has been associated with the development of diabetes and hypertriglyceridemia [8, 9], which are also associated with DSP [7, 10, 11]. These cohorts differ substantially from the vast majority of people presently receiving PIs in sub-Saharan Africa; for example, African cohorts are young, predominantly women, with very low hepatitis C co-infectivity and intravenous drug abuse [12]. There are no data on the frequencies of DSP and metabolic risk factors in African cohorts on PIs. Here we assessed the association between DSP and metabolic factors in a community-based South African cohort on second-line ritonavir/lopinavir-based ART. Secondly, we compared the frequencies of DSP to that of a nested-group who had remained on first-line stavudine-containing ART and were matched for a similar duration of ART.

## Results

### Study population characteristics

The ritonavir/lopinavir-group (n = 86) had a median age of 36 years and the majority were women (84 %). Their median current CD4+ count was 480 cells/μL although the nadir pre-ART CD4+ counts were frequently <100 cells/μL (Table 1). Overall, these participants had been on the government-sponsored ART program for a median of 36 months and their median exposure to ritonavir/lopinavir was 18 months (IQR 10–28 months). In most cases (55 %) second-line ART was started after virological failure of first-line regimens and in 26 % it was started due to side effects on first-line ART including symptomatic hyperlactataemia (presence of nausea, vomiting, abdominal pain or weight loss and plasma lactate >2.5 mmol/L) (n = 5), “neuropathy” (n = 4), drug-induced hepatitis (n = 4), lipodystrophy (n = 4) and nevirapine-associated rash (n = 3). Pregnancy resulted in a switch to PIs in 7 and in 12 % we were unable to determine the reason for changing from first-line ART. All participants had previous or current d-drug exposure including stavudine (90 %) and/or didanosine (64 %). First-line ART at the time of this study comprised stavudine or zidovudine, lamivudine and nevirapine or efavirenz. Second-line ART comprised stavudine or didanosine (if stavudine was a first-line agent), lamivudine and ritonavir/lopinavir-group. No individuals were taking statins or concomitant oral hypoglycemic agents.

**Table 1:** Clinical and laboratory characteristics of DSP and symptomatic DSP in the ritonavir/lopinavir-group (n = 86)

	Normal value	No DSP (*n* = 19)	DSP (*n* = 67)	P value	Symptomatic DSP (*n* = 41)	P value
Age, years		33 (27–36)	37 (33–43)	*0.007*	37 (34–42)	*0.007*
Female, *n* (%)		17 (89)	55 (82)	0.45	33 (80)	0.39
Alcohol^a^, *n* (%)		8 (42)	21 (32)	0.41	14 (34)	0.55
Previous TB, *n* (%)		12 (63)	49 (74)	0.35	35 (85)	0.059
Total d-drug exposure, mo.		26 (12–36)	24 (18–38)	0.67	22 (15–39)	0.53
Stavudine exposure, mo.		15 (11–34)	15 (10–22)	0.33	15 (8–25)	0.32
Didanosine exposure, mo.		19 (12–21)	13 (7–23)	0.24	13.5 (7–25.5)	0.29
LPV/r exposure, mo.		21 (12–35)	18 (10–26)	0.08	18 (10–26)	0.24
CD4 nadir, cells/µL		84 (54–180)	96 (37–122)	0.28	68 (25–120)	0.13
CD4 current, cells/µL		576 (467–726)	418 (251–610)	*0.030*	472 (251–752)	0.15
Viral load at ART initiation		184,563 (30,438–244,623)	96,645 (36,820–241,495)	0.54	108,572 (36,858–280,000)	0.92
Height (m)		1.64 (1.63–1.66)	1.61 (1.56–1.65)	0.15	1.62 (1.58–1.68)	0.45
Weight (kg)		95 (70–98)	64 (57–76)	*<0.001*	68.5 (59.9–82)	*0.008*
Body mass index (kg/m^2^)	<25	34.5 (26.0–38.5)	25.1 (23.3–29.5)	*0.005*	26.1 (23.26–30.29)	*0.018*
Waist circumference (cm)		103.7 (92.5–109.5)	85.0 (78.5–93.5)	*0.001*	87.0 (78.5–99)	*0.017*
Systolic blood pressure (mmHg)		110 (103–125)	107 (102–109)	0.13	107 (102–119)	0.15
Pre-diabetes, *n* (%)		5 (26)	22 (33)	0.59	11 (27)	0.97
Diabetes, *n* (%)		2 (11)	6 (9)	0.84	4 (10)	0.93
Total cholesterol (mmol/L)	<5.17	4.58 (4.13–5.49)	4.33 (3.69–5.35)	0.56	3.98 (3.66–5.05)	0.20
HDL (mmol/L)	>1.03	0.93 (0.75–1.20)	0.99 (0.80–1.17)	0.79	0.98 (0.76–1.17)	0.99
LDL (mmol/L)	<2.60	3.18 (2.66–3.39)	2.56 (2.18–3.14)	0.21	2.46 (2.12–3.00)	0.06
Triglycerides (mmol/L)	<1.70	1.23 (0.97–1.57)	1.36 (0.89–1.92)	0.91	1.29 (0.93–1.79)	0.79
Fasting lactate (mmol/L)	<1.5	2.3 (1.5–2.9)	2.1 (1.7–2.8)	0.73	2.2 (1.7–3.0)	0.81

All continuous variables shown as median value (inter quartile range). Pre-diabetes defined as fasting plasma glucose (FPG) ≥5.6 mmol/L but <7.0 mmol/L or 2-h plasma glucose during the oral glucose tolerance test [OGTT] ≥7.8 mmol/L but <11.1 mmol/L. Diabetes defined as FPG ≥7.0 mmol/L or 2-h OGTT ≥11.1 mmol/L

*DSP* distal sensory polyneuropathy, defined by the presence of ≥1 neuropathic sign, *symptomatic DSP* DSP in the presence ≥1 neuropathic symptom, *Mo*. months, *LPV/r *ritonavir/lopinavir

Italic values indicate statistically significant P values

^a^Any alcohol use in the past 12 months. P values for DSP and symptomatic DSP derived by comparing with no DSP

### Frequencies and risk factors of distal sensory polyneuropathy

DSP was present in 67 (78 %) of individuals in the ritonavir/lopinavir-group and symptomatic DSP in 41 (48 %) (Table 1). Pain and/or paresthesiae were more frequent complaints than numbness (78 vs. 66 %) and the symptom severity grade was most often classified as moderate (Additional file 1: Table S1). Altered reflexes, either reduced or absent, was the most frequent neuropathic findings in both DSP (73 %) and symptomatic DSP (85 %), altered distal vibration sense in 63–64 % of subjects and impaired distal pain sensibility in 37–39 % of those with DSP and symptomatic DSP, respectively. Mild/moderate weakness of the toes/ankles was present in ≤25 %.

Those with DSP were older (37 vs. 33 years, p = 0.007) with lower current CD4+ counts (418 vs. 576 cells/µl, p = 0.030) although their nadir CD4+ counts were similar to those without DSP. In addition, subjects with DSP compared to those without DSP had lower macro-nutritional indicators including bodyweight (64 vs. 95 kg; p < 0.001), body mass index (BMI) (25.1 vs. 34.5 kg/m^2^; p = 0.005) and waist circumference (85.0 vs. 103.7 cm; p = 0.001). Obesity (BMI >30 kg/m^2^) was present in 22 % of the DSP group and 54 % of those without DSP (p = 0.009). A multivariate logistic regression model found none of these factors independently associated with DSP although a trend remained with those older than 36 years (p = 0.08; Table 2).

**Table 2:** Multivariate analysis of risk factors for DSP and symptomatic DSP in the ritonavir/lopinavir-group

	Univariate analysis		Multivariate analysis	
	Odds ratio (95 % CI)	P value	Odds ratio (95 % CI)	P value
DSP				
Age >36 years				
Yes	*3.9 (1.26–12.08)*	*0.018*	7.0 (0.88–55.65)	0.08
No	1			
BMI < 30 kg/m^2^				
Yes	*6.19 (1.56–24.47)*	*0.009*	0.38 (0.03–5.37)	0.47
No	1			
Waist circumference increased^a^				
Yes	*0.18 (0.54–0.60)*	*0.005*	1.33 (0.11–15.5)	0.82
No	1			
Current CD4 count <250 cells/μL				
Yes	5.88 (0.73–47.58)	0.09	0.43 (0.03–6.20)	0.54
No	1			
Symptomatic DSP				
Age >36 years				
Yes	*4.38 (1.32–14.50)*	*0.016*	*7.39 (1.07–50.77)*	*0.042*
No	1			
BMI <30 kg/m^2^				
Yes	*4.14 (1.00–17.05)*	*0.049*	0.38 (0.03–5.62)	0.38
No	1			
Waist circumference increased^a^				
Yes	*0.23 (0.06–0.81)*	*0.023*	1.86 (0.12–29.60)	0.66
No	1			

*DSP* distal sensory polyneuropathy, defined by the presence of ≥1 neuropathic sign, *symptomatic DSP* DSP in the presence ≥1 neuropathic symptom, *BMI* body mass index

^a^Defined as a waist circumference of >88 cm for women and >102 cm for men

Symptomatic DSP also showed significant associations with age (p = 0.007) and lower macro-nutritional indicators including bodyweight (p = 0.008), BMI (p = 0.018) and waist circumference (p = 0.017), but on multivariate analyses only age remained as an independent risk factor (p = 0.042; Table 2). A history of previous tuberculosis infection(s) showed a trend towards associating with symptomatic DSP (p = 0.059).

### Metabolic factors and distal sensory polyneuropathy

Dysglycemia was found in 41 % of the ritonavir/lopinavir-group although neither the fasting glucose nor OGTT values associated with DSP or symptomatic DSP (Table 1). Hypertriglyceridemia, defined as fasting triglycerides ≥1.7 mmol/L (≥150 mg/dl), was found in 29 %, but fasting triglyceride levels did not show an association with neuropathy status (Table 2). Low density lipoprotein (LDL) levels were not associated with DSP but individuals with symptomatic DSP had a trend (p = 0.06) towards lower values.

### Protease inhibitors + d-drugs vs. d-drugs only and the risk of distal sensory polyneuropathy

As reports from high-income countries previously alluded to an additional risk of PIs and DSP we next compared the frequencies of DSP in this ritonavir/lopinavir-group who had prior exposure to NRTI-drugs as first-line therapy, and a nested NRTI-group remaining on first-line ART but matched for overall d-drug exposure (Table 3). Although the two groups had similar age distributions (p = 0.57) they differed in several aspects; the ritonavir/lopinavir-group had proportionately more women (p = 0.019), lower nadir CD4+ counts (p = 0.044) and higher lactate levels (p = 0.013). The ritonavir/lopinavir-group was taller (1.62 vs. 1.51 m; p < 0.0001) with lower BMI values (p = 0.036) but they had larger waist circumference values (p = 0.029). The ritonavir/lopinavir-group had higher fasting triglyceride levels (1.29 vs. 1.10 mmol/L, p = 0.021) and more participants met criteria for hypertriglyceridemia (29 vs. 12 %, p = 0.031). However, the proportion of participants with DSP was lower in the ritonavir/lopinavir-group (78 %) when compared with the nested d-drug only group (94 %; odds ratio = 0.22; 95 % CI 0.07–0.6; p = 0.002) and the frequencies of symptomatic DSP were similar in both groups (48 vs. 53 %, p = 0.49).

**Table 3:** Demographics and clinical features in the ritonavir/lopinavir-group compared to the NRTI-only nested control-group

	Normal value	Ritonavir/lopinavir	D-drug only^a^	P value
		n = 86	n = 85	
Age, years		35 (32–41)	36 (30–42)	0.57
Female, *n* (%)		72 (84)	58 (68)	*0.019*
Previous tuberculosis, *n* (%)		61 (72)	58 (74)	0.71
Alcohol usage, *n* (%)^b^		29 (34)	21 (25)	0.18
Period on d-drugs, months^c^		24 (16–37.5)	23 (18–28)	0.33
DSP, *n* (*%*)		67 (78)	80 (94)	*0.004*
Symptomatic DSP, *n* (*%*)		41 (48)	45 (53)	0.49
Height (m)		1.62 (1.57–1.65)	1.51 (1.44–1.57)	*<0.001*
Weight (kg)		67 (59–85)	66 (58–75)	0.10
Body mass index	<25	26.0 (23.6–30.9)	30.0 (24.0–34.0)	*0.036*
Waist circumference (cm)^d^		87.8 (79.3–100.3)	83.8 (77.5–92.0)	*0.029*
Systolic BP (mmHg)		108 (102–119)	108 (100–116)	0.81
CD4 current (cells/µL)		489 (291–665)	406 (296–564)	0.49
CD4 nadir (cells/µL)		96 (37–132)	120 (55–160)	*0.044*
Viral load at start of ARV		102,268 (36,820–241,495)	53,000 (12,000–360,000)	0.51
Pre-diabetes, *n* (%)		27 (32)	27 (39)	0.24
Diabetes, *n* (%)		8 (9)	3 (4)	0.35
Total cholesterol (mmol/L)	<5.17	4.42 (3.75–5.41)	4.31 (3.45–4.83)	0.11
HDL (mmol/L)	<1.03	0.96 (0.76–1.17)	0.97 (0.79-1.18)	0.90
Triglycerides (mmol/L)	<1.70	1.29 (0.93–1.82)	1.10 (0.80–1.47)	*0.021*
Hypertriglyceridemia, *N* (%)	>1.70	25(29)	10 (12)	*0.031*
Fasting lactate (mmol/L)	<1.5	2.2 (1.7–2.9)	1.8 (1.4–2.4)	*0.013*
Hyperlactatemia, *N* (%*)*	>2.5	32 (39)	20 (25)	0.06

The results of all continuous variables are shown as median (inter quartile range). *DSP* distal sensory polyneuropathy as defined by ≥1 neuropathic sign. Symptomatic DSP is defined as ≥1 neuropathic sign and symptom. Prediabetes defined as fasting plasma glucose (FPG) ≥5.6 mmol/L but <7.0 mmol/L or 2-h plasma glucose during the oral glucose tolerance test (OGTT) ≥7.8 mmol/L but <11.1 mmol/L. Diabetes defined as FPG ≥7.0 mmol/L or 2-h plasma glucose during the OGTT ≥11.1 mmol/L

^a^The nested controls were extracted from a cohort [2] as outlined in Methods

^b^Any alcohol use in the prior 12 months

^c^Defined as the sum of stavudine and didanosine exposure times

^d^Men <90 cm, women <84 cm. P value reflects the comparison of those exposed to ritonavir/lopinavir vs. the matched controls who did not have ritonavir/lopinavir exposure

## Discussion

We present the first cross-sectional evaluation of the frequency and risk factors of DSP in a HIV-infected African cohort receiving the protease inhibitor, ritonavir/lopinavir, as second-line ART for approximately 12–24 months. The frequency of DSP (using the non-stringent but standard definition) was high at 78 %, but importantly, almost half of the cohort (48 %) had symptomatic DSP. In contrast, after a similar period of ART, North American cohorts showed DSP frequencies of ≈32–50 % and symptomatic DSP of ≈20 % [7]. Interestingly, we found that a comparator d-drug only group matched for the duration of d-drug exposure as first-line ART, showed similar frequencies of symptomatic DSP. This suggests that the PI-regimen was not conferring additional risk to developing DSP. An important aim of this study was to investigate the association of DSP and metabolic factors given the potential of ritonavir/lopinavir to induce dysglycemia and dyslipidemia [8]. However, we found no associations between these metabolic factors and DSP or symptomatic DSP.

This African cohort had relatively advanced HIV-disease when starting ART, and after 2–3 years on ART, older age was the only independent risk factor for symptomatic DSP. Advancing age remains the most consistent factor associated with DSP and symptomatic DSP in both ART-naïve and ART-exposed cohorts irrespective of the ART regimen [1, 2, 7, 13, 14]. In many HIV-infected cohorts comprising mostly of men, height has been associated with symptomatic DSP [14, 15]. We have not found this association in either a previous [2], or this current cohort, both of which consisted mostly of indigenous black South African women. Similar to previous reports from Africa, a previous history of tuberculosis was again shown to segregate with symptomatic DSP [2, 16]. There are multiple reasons why HIV/TB co-infected individuals might be at greater risk of DSP including treatment with isoniazid, micronutrient deficiencies including inadequate pyridoxine supplementation and the cumulative oxidative stress of HIV/TB co-infection [16–19].

The broader applicability of our results in the current era of ART has several constraints; firstly, similar to many HIV-infected cohorts in sub-Saharan Africa and differing from the developed world, few individuals in this cohort are older than 40 years. Our participants are predominantly young women and although HIV-infection is expected to accelerate ageing and impact on the metabolic syndrome it may still be too early for the metabolic manifestations of ART. In contrast to our findings, cross-sectional North American studies found elevated random [11] and fasting [10] triglyceride levels to be associated with DSP but these cohorts consisted mostly of older men.

## Conclusions

Despite high frequencies of DSP and symptomatic DSP among South Africans receiving ritonavir-boosted lopinavir (for a median of 18 months) following prior exposure to stavudine-containing ART, our result show that this protease inhibitor, was not associated with additional risk of developing DSP. Although we found no association between DSP and metabolic factors after at least 12 months of exposure to protease inhibitors in this relatively young cohort of mostly black women, longer-term observational studies are required as this population ages.

## Methods

### Study population and methods

The participants for this cross-sectional study were recruited between 2008 and 2010 from two community healthcare centers in Cape Town, South Africa. The South African governmental HIV treatment program at the time, used the PI, ritonavir/lopinavir as part of their second-line ART in combination with two NRTI-class drugs. Eligible subjects had to be on ritonavir/lopinavir-containing second-line ART for ≥6 months. Participants were excluded if they had: a history of diabetes mellitus; active opportunistic infection; severe diarrhea (six stools/day); tuberculosis <1 month of commencing treatment; received glucocorticoid therapy within the past 6 months; pregnant; known with renal failure; neurological disorder confounding the assessment of neuropathy. The study was approved by the University of Cape Town Research Ethics Committee.

After signing informed consent, study participants were assessed by one of two trained doctors using the Brief Peripheral Neuropathy Screen (BPNS) and a revision of the modified Total Neuropathy Score (TNSr) as previously described [2, 20]. Supervision of examination procedures by a neurologist was frequent and ongoing throughout the study period. The definition for DSP included the symmetrical distal onset of ≥1 neuropathic sign in the feet: reduced/absent reflexes, impaired distal vibration or pin sensibility. Symptomatic DSP referred to DSP with symmetrical neuropathic symptoms: burning pain, paresthesiae or numbness. Symptom severity was assessed using a visual numerical scale ranging between 0 and 10 and then graded as mild (1–3), moderate (4–6), severe (7–8) and very severe (9–10). Blood pressure and anthropometry (weight, BMI, waist circumference), as well as an oral glucose tolerance test (OGTT) and fasting lipid levels were performed as previously described [21]. Pre-diabetes was defined as fasting plasma glucose (FPG) ≥5.6 mmol/L but <7.0 mmol/L or 2-h OGTT plasma glucose ≥7.8 mmol/L but <11.1 mmol/L. Diabetes was defined as FPG ≥7.0 mmol/L or 2-h OGTT ≥11.1 mmol/L. Demographic information including drug history was obtained by questionnaire and folder review.

For comparison, a nested-group on first-line ART (containing the d-drug, stavudine) were selected from a previously described cross-sectional cohort [2] who were recruited from the same clinics, according to the same inclusion/exclusion criteria and underwent evaluations according to the same protocol by the same study team. Although there was some overlap in the study period, this second-line ART study started at least 12 months after the first-line study. The groups were matched for duration of ART and d-drug exposure in the following manner: all subjects on first-line ART (n = 216) and shorter total d-drug exposure times were systematically censored until there was no statistical difference in the exposure times between the nested d-drug cohort and the ritonavir/lopinavir-group. This point was reached when d-drug exposure ≥14 months and included 85 subjects.

### Statistical analysis

For univariate analyses continuous variables were assessed by Shapiro–Wilk testing and non-parametric data was transformed by the most appropriate transformation selected from a ladder of powers of transformations. Student *t* tests were used for parametric continuous data, Wilcoxon rank-sum tests for non-transformable non-parametric data and Chi square tests for categorical data. Statistical significance was set at the 0.05 level (two-sided). Certain continuous variables were categorized based on clinically relevant cutoff values. Variables showing significant associations were systematically included in a step- wise multivariate logistic regression model. Spearman correlates were used to investigate the relationship between the variables included in each model. All data analyses were performed using STATA/IC 11.

## Additional file

10.1186/s12981-015-0073-8-S1.pdf The neuropathy characteristics of DSP and symptomatic DSP in the ritonavir/lopinavir-group.

## Abbreviations

ART: antiretroviral therapy
PI: protease inhibitors
DSP: distal sensory polyneuropathy
IQR: interquartile range
OGTT: oral glucose tolerance test
FPG: fasting plasma glucose
BMI: body mass index
