# Supplementary material for: Sensory neuropathy and metabolic risk factors in human immune deficiency virus infected South Africans receiving protease inhibitors
Source: AIDS Res Ther. 2015 Sep 23;12:30. doi: 10.1186/s12981-015-0073-8 (PMC4580116; doi:10.1186/s12981-015-0073-8)
Supplement: Supplementary file 1 — 10.1186/s12981-015-0073-8-S1.pdf The neuropathy characteristics of DSP and symptomatic DSP in the ritonavir/lopinavir-group. [file 12981_2015_73_MOESM1_ESM.pdf]

Additional Table:

**The neuropathy characteristics of DSP in the ritonavir/lopinavir-group**				
	**No DSP**<br>**n = 19 (22%)**	**DSP**<br>**n = 67 (78%)**	**DSP**	
			**Asymptomatic**<br>**n = 26 (30%)**	**Symptomatic**<br>**n = 41 (48%)**
No symptoms, *N (%)*	14 (74)	26 (39)		
Neuropathic symptom, *N (%)*				
Pain	4 (21)	32 (48)		32 (78)
Paresthesia	4 (21)	32 (48)		32 (78)
Numbness	2 (11)	27 (40)		27 (66)
Neuropathic sign, *N* (%)				
Hypo/areflexia		54 (80)	19 (73)	35 (85)
Impaired vibration sense		43 (64)	17 (65)	26 (63)
Impaired pin sense		25 (37)	9 (35)	16 (39)
Mild/moderate weakness*	1 (5)	13 (19)	3 (10)	10 (25)

Legend: DSP = the presence of at least one of the following neuropathic sign: reduced or absent ankle reflexes, impaired vibration or impaired pin sensation. *Defined as reduced dorsi-flexion strength in ankle/toes. Weakness was graded as mild, moderate, severe or paralysis
